# A Component Formula of Chinese Medicine for Hypercholesterolemia Based on Virtual Screening and Biology Network

**DOI:** 10.1155/2018/1854972

**Published:** 2018-06-28

**Authors:** Xiaoqian Huo, Fang Lu, Liansheng Qiao, Gongyu Li, Yanling Zhang

**Affiliations:** School of Chinese Pharmacy, Beijing University of Chinese Medicine, Beijing 100102, China

## Abstract

Hypercholesterolemia is a risk factor to atherosclerosis and coronary heart disease II. The abnormal rise of cholesterol in plasma is the main symptom. Cholesterol synthesis pathway is an important pathway of the origin of cholesterol, which is an essential pathway for the therapy of hypercholesterolemia. The 3-hydroxy-3-methylglutaryl-coenzyme A reductase (HMG-CoA reductase), squalene synthase (SQS), and sterol regulatory element binding protein-2 (SREBP-2) are closely connected with the synthesis of cholesterol. The inhibition of these targets can reduce the cholesterol in plasma. This study aimed to build a component formula including three Traditional Chinese Medicines (TCM) components with the inhibition activity of these targets by using virtual screening and biological network. Structure-based pharmacophore models of HMG-CoA reductase and SQS and ligand-based pharmacophore model of SREBP-2 were constructed to screen the Traditional Chinese Medicine Database (TCMD). Molecular docking was used for further screening of components of HMG-CoA reductase and SQS. Then, metabolic network was constructed to elucidate the comprehensive interaction of three targets for lipid metabolism. Finally, three potential active compounds were obtained, which are poncimarin, hexahydrocurcumin, and forsythoside C. The source plants of the compounds were also taken into account, which should have known action of lowering hyperlipidemia. The lipid-lowering effect of hexahydrocurcumin was verified by experiment in vitro. The components that originated from TCMs with lipid-lowering efficacy made up a formula with a synergistic effect through the computer aid drug design methods. The research provides a fast and efficient method to build TCM component formula and it may inspire the study of the explanation of TCM formula mechanism.

## 1. Introduction

Hypercholesterolemia, a kind of hyperlipidemia, is the abnormal rise of serum cholesterol, which is an induced factor to atherosclerosis and coronary heart disease [1, 2]. The cholesterol synthesis pathway generates most of cholesterol in the human body, which provides a significant approach for antihypercholesterolemia. So the targets in the cholesterol synthesis pathway are always hot spots for new lipid-lowering drug development. There are multiple targets in the synthesis pathway. Besides, some targets have been important targets for the drug design because of better pesticide effect. 3-Hydroxyl-3-methylglutaryl-coenzyme A reductase (HMG-CoA reductase) is one of the most commonly used targets in the therapy of the hyperlipidemia, which is the limit enzyme of cholesterol synthesis. As the inhibitor of HMG-CoA reductase, statins provided many benefits for patients including effective reduction of TC, TG, low-density lipoprotein cholesterol (LDL-C) levels, and neovascularization and lowering cerebral injury with curative effect and security [3–6]. Squalene synthase (SQS) was another key enzyme in the cholesterol synthesis, which can catalyze the biosynthesis of the key cholesterol precursor, squalene [7]. The design of SQS inhibitor has never stopped. Some SQS inhibitors have favorable therapeutic effect in reducing cholesterol [8, 9]. Because of the protein-protein interaction between HMG-CoA reductase and SQS, the inhibitor of the SQS can be a complementary drug of the HMG-CoA reductase. The research provided evidence of SQS inhibitors as a monotherapy and adjunctive therapy for the patients who cannot achieve a satisfied cholesterol target goal [10]. The coadministration with statins may decrease statin-induced myotoxicity [11, 12]. New SQS inhibitors have been in continuous research [13]. Sterol regulatory element binding protein-2 (SREBP-2), a member of the SREBP family (SREBPs), is an important cholesterol regulator in cells that can directly activate the expression of more than 30 genes including HMG-CoA reductase, SQS, and other important enzymes and regulatory factors in the lipid metabolism pathway [14–16]. It has been discovered that the inhibitors of SREBP-2 can decrease cholesterol level and have well application prospect in the treatment of hypercholesterolemia. The targets were chosen including two important enzymes in the synthesis pathway and an important regulator. The protein-protein interaction between the targets can get more satisfied antihyperlipidemia curative effect [17, 18]. The Traditional Chinese Medicines (TCM) were considered as a source of inspiration of new drugs [19]. The TCM were a complex system that includes multiple components. Components take synergistic effect by acting on different targets for enhancing curative effect and reducing the side effects [20]. So, microconstituents in TCM can play the same or a better role of the megadose of single component. The formula was a traditional drug combination method that combines Chinese medicines with different effect under the guidance of TCM [21]. Component formula of the Chinese medicine was a combination of the TCM components or extracts. Component formula covers the shortage of single-ingredient drug and provides the benefit such as the clear action targets and mechanism of herbal pharmacology. The component formula of the Chinese medicine is a theoretical innovation in the Chinese materia medica theory.

This research hopes to build a component formula based on the computer aid drug design (CADD). Pharmacophore and molecular docking models were used for the virtual screening of the TCM component with potential inhibition activity of HMG-CoA reductase, SQS, and SREBP-2. A network of these three targets was built to show the synergistic effect between three targets, which provided the mechanism of action for the component formula. Besides, the TCM components were chosen under the consideration of TCM theory.

Pharmacophore and molecular docking models were built for the virtual screening. The components with potential activity of chosen targets were chosen. To expound the synergistic effect between the components, a network based on three targets was built. Pharmacophore and molecular docking were two important methods in the virtual screening. Pharmacophore model, which was an important method of molecular modeling for virtual screening, has been widely used to search the potential active compounds [22, 23]. Ligand-based pharmacophore model is generated by collecting important atoms and atoms groups of active ligands, which shows the necessary features of active ligands. Galahad is one of the most frequently used methods of ligand-based pharmacophore [24]. Receptor-based pharmacophore models are based on the known three-dimensional (3D) structure of targets, which can reflect the spatial relationships between protein structure and ligand in active site. Structure-based pharmacophore (SBP) method is a kind of receptor-based pharmacophore model. In the present study, pharmacophore models of HMG-CoA reductase inhibitors and SQS inhibitors were built by SBP, respectively, while pharmacophore models of SREBP-2 inhibitors were built by Galahad because of lacking protein structures. The best pharmacophore models of each target were selected and used to search the Traditional Chinese Medicine Database (TCMD, vision 2009) [25, 26]. Then, virtual screening results of HMG-CoA reductase and SQS were further screened by molecular docking. Molecular docking is another important method in CADD, which can demonstrate the interaction between ligand and the receptor. The information of the interaction was valuable in the drug design. Recently, biological network provides a new method for complex system research. Metabolic network, an important type of biological network, can reflect the interaction among compounds, enzymes, and regulatory factors involved in the metabolic process for the analysis. The application of metabolic network provides a bridge to study on the mode of action of TCM, which has been widely used for thousands of years by taking advantage of “multiple ingredients and multiple targets” [27–29]. In this paper, one metabolic network model was constructed for analyzing the mechanism of interaction among HMG-CoA reductase, SQS, and SREBP-2 to explain how effectiveness was enhanced by drug combination through the interaction among the targets.

This research hopes to build a component formula, which has obvious advantages such as exact dose and relatively clear mechanisms of action. This work combined the CADD methods and TCM theory for a component formula. The CADD can effectively screen component in a short time and provided the information of targets and interaction of the component for further study. Network elucidates the synergistic effect between the targets, or it may aid the discovery of potential targets and the mechanism analysis. Then, the source plant can diminish the range of suitable components that are more likely to have activity.

In summary, the virtual screening is a powerful method for searching active compounds from TCM and the mechanism of complex effect of multicompounds can be clearly illuminated based on biological network. The component formula with clear material foundation and certified mechanism can be formed through the combination of potential active compounds with different targets' inhibition activity. The article aims to provide a clue to new component formula design and the study of mechanism of TCM component formula based on virtual screen and biological network.

## 2. Materials and Methods

### 2.1. Construction of Pharmacophore

The pharmacophore models of HMG-CoA reductase and SQS were constructed by “Receptor-Ligand Pharmacophore Generation Protocol” based on the crystal structures of proteins that were obtained from the Protein Data Bank (http://www.rcsb.org/pdb/home/home.do) in the Discovery Studio (DS, vision 4.0) [30]. By entering the “HMG-CoA reductase” and “Squalene synthase” as the search terms in the PDB, the structures with high resolution and known active ligands were chosen to be applied in the research. The information of structures was shown in Table 1.

The conformations for all compounds were generated by the BEST conformation generation method. Active site was the site of the ligand combined with the structure and the location of the pharmacophore. Active site was searched automatically. All crucial features, including Hydrogen Bond Acceptor (A), Hydrogen Bond Donor (D), and Hydrophobic (H), would be mapped in the active sites. Furthermore, A, D, and H features were clustered automatically and the most crucial features were chosen to build pharmacophore model by employing Cluster Current Features protocol. Besides, Ev (Exclusion volume) spheres were added to the inaccessible areas in active site.

**Figure 1:**
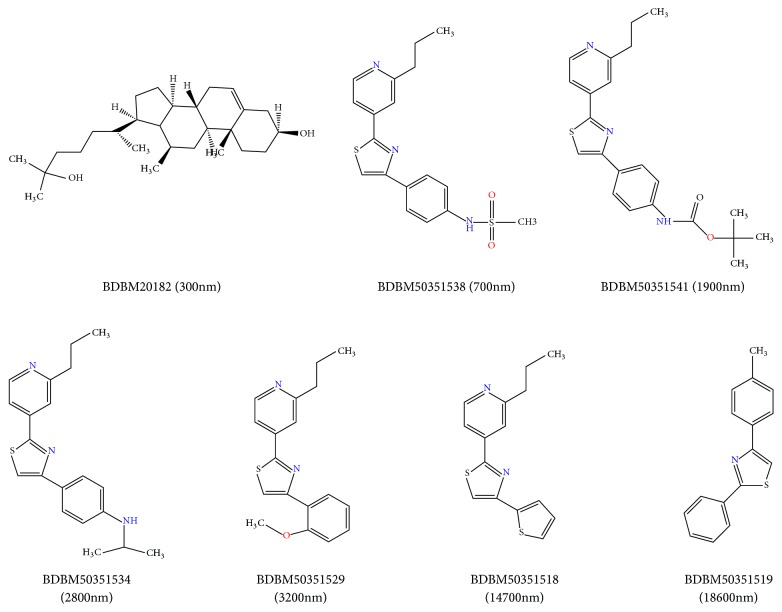
The structures and IC_50_ values of the compounds in the training set for SREBP-2 pharmacophore modeling.

Because of lacking the whole structure of SREBP-2, ligand-based pharmacophore method, Galahad, was applied to construct SREBP-2 inhibitor pharmacophore models [31]. Seven compounds with the known activity of SREBP-2 were chosen from the Binding database (http://www.bindingdb.org) as the training set to construct the pharmacophore models. The structure and the IC_50_values of the compounds in the training set were shown in Figure 1. The force field was set to Tripos and the charge was set to Gasteiger-Huckel atomic partial charges. The energy was minimized by the Powell method. The optimal set of the conformations was fixed as rigid bodies in Cartesian space. Then twenty models were generated by the genetic algorithm with different features, conformations, and overlay of the molecules [32, 33].

### 2.2. Pharmacophore Validation

Test set, including active compounds and inactive compounds, is used to assess the discrimination capacity of pharmacophore models. The numbers of the compounds in the test sets were shown in Table 2. Active compounds were obtained from the Binding database, while the inactive compounds were chosen from the MDDR (MDL Drug Data Report: Version 2007.2).

Hit ratio of active compounds (*HRA*), identification of effective index (*IEI*), and comprehensive appraisal index (*CAI*) values were employed to evaluate the SBP models. The high* HRA* shows a great ability of pharmacophore model to identify active compounds in the test set. The high* IEI* means an outstanding ability to identify active compounds from inactive compounds.* CAI* is calculated to evaluate the models comprehensively [34, 35].

The SBP model with highest* CAI* and* IEI* would be chosen as the best pharmacophore model to screen the TCMD. The best pharmacophore models built by Galahad could be evaluated by both test set result and the internal evaluation, which was selected by the following three criteria: (1) the model should be in high* IEI *and* CAI* values; (2) the model needs to have lower energy; (3) the model needs to have high specificity. The best pharmacophore model would be used to screen the TCMD.

### 2.3. TCMD Screening

The best pharmacophore models of three targets were utilized as queries to search the TCMD, which include 233,033 compounds from the Traditional Chinese Medicine. The “flexible database search” was carried out in virtual screening process. FIT value of each compound was calculated to assess the mapping quality of the compounds. The higher FIT value suggested a better mapping between the compounds and the pharmacophore model. Then the hit compounds would satisfy Lipinski's rules. Finally, the compounds with drug-like character and potential inhibited activity were listed for further docking research.

### 2.4. Molecule Docking

The crystal structure 3ASX and 1HWL were used for molecular docking of HMG-CoA reductase and SQS. The binding site was the same to pharmacophore model. LibDock and CDOCKER were preliminarily selected in this work. To choose a more appropriate algorithm, the initial compounds FBI and D99 were extracted from the binding site and redocked into the structure for calculating the RMSD value. The RMSD was used to evaluate a rationality of the algorithms and docking parameter settings. In general, RMSD of less than 2.00Å indicated that the algorithm could reproduce the observed binding mode for the initial compound [36, 37]. A smaller RMSD value means that the docking method better fits the system. The compounds of each target from the pharmacophore results were docking into the protein structure by the same methods of the initial compounds. Scoring functions were used to evaluate the results. The compounds with higher scores may have better interaction.

### 2.5. Metabolic Network Construction

The pathways of the three targets were obtained from the Reactome database (http://www.reactome.org) [38]. Then the pathways were imported into the Cytoscape (vision 2.8.3) [39, 40]. The construction of the network was completed in the Cytoscape by merging the pathways of three targets. Then the isolated nodes and self-loop edges would be removed and the repeated sides and points were deleted.

The parameters were calculated to certify the stability and fault tolerance of the network. Network connectivity is the character for evaluating the connected condition of network. The network has high antidestroying ability when the connectivity value reaches 100%. Scale-free property of network is evaluated by degree distribution. Network has a scale-free property when the degree distribution of the network obeyed power-law distribution, which has high fault tolerance. Barabasi-Albert model (BA model) put forward by Barabasi and Albert [41] pointed the standard of the scale-free network.

(1) $$\begin{array}{c} Y=aX^{b} \end{array}$$

In generation, b<3 is the criterion to evaluate whether the network is in scale-less property. The networks can be applied intuitively to show the interaction among the three targets and the mechanism of formula synergistic effect.

### 2.6. The Design of TCM Component Formula

The component was chosen by the following two criteria: (1) component of HMG-CoA reductase and SQS should have a high rank of the sum of the FIT value ranks and docking score ranks of the compounds. The sum can evaluate the activity of the compounds. The compound that has a higher rank may have a higher activity; (2) the source plants of HMG-CoA reductase, SQS, and SREBP-2 should have a known lipid-lowering action or form a formula related to lipid-lowering. The source plants showed the traditional use in the TCM, which provide a clue of the activity of component. Network of the targets was built for the analysis of synergistic effect between the components. The related targets were explored in the network, which inspired other effects or the mechanism of action of the formula.

### 2.7. The Activity of TCM Compounds

The activity of TCM compounds was verified on the cell. HepG2 cell lines were used for the MTT assay and activity assays. Cells were cultured at 1×104 cells/well in 96-well culture plate. The cells were adhered to and treated with different concentrations of the targeted compounds after 24 h and cultured at 37°C in the atmosphere of 5% CO_2_. Then, MTT in 0.5 mg/ml was added to each well after the supernatants were discarded and incubated at 37°C in 5% CO_2_ for an additional 4 h. After that, 150 *μ*L of formazan in dimethyl sulfoxide (DMSO) was added after the MTT was removed. And then the plates were shaken for 5 min at low speed. Cell proliferation was evaluated by measuring the absorbance at 570 nm using ELISA Plate Reader. The IC_50_ values were calculated by SPSS Statistics 17.0.

## 3. Results and Discussion

### 3.1. Pharmacophore Generation

Two pharmacophore models of HMG-CoA reductase were built in this study. The models contained hydrophobic features and hydrogen bond acceptor feature. SQS pharmacophore models were composed of the hydrophobic features, hydrogen bond acceptor features, and hydrogen bond donor feature. Ev features were added to each pharmacophore where in the protein structure the compounds cannot approach. The number of the Ev features in HMG-CoA reductase was fourteen and the number in the SQS was eighteen. Twenty models of SREBP-2 were generated [42, 43]. The results of three of them were shown in Table 2. The pharmacophore models of SREBP-2 contained hydrophobic features, hydrogen bond acceptor features, and hydrogen bond donor feature.

### 3.2. Pharmacophore Validation

**Figure 2:**
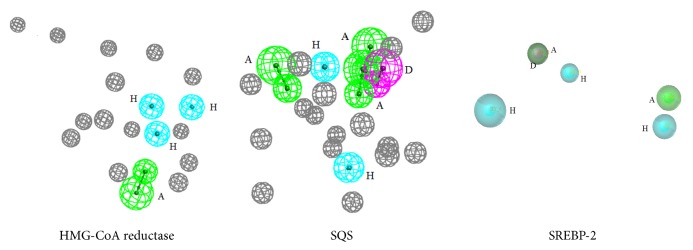
The best pharmacophores of each target.

The pharmacophore validation results were shown in Table 2. M-HMG-CoA reductase-1 and M-SQS-1 with the highest IEI and CAI values were chosen as the best models to screen the TCMD. M-SREBP-2 has the least energy and high specificity, IEI, and CAI value. So it was chosen to screen the TCMD. The best pharmacophore was shown in Figure 2.

**Figure 3:**
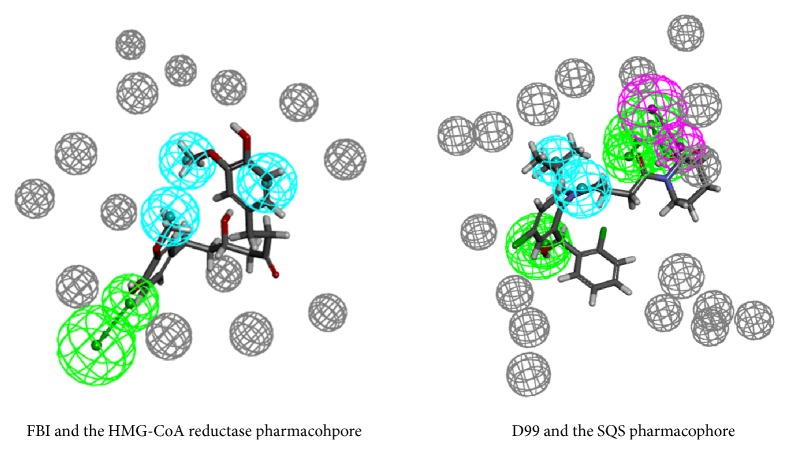
The mapping results of each initial compound.

The M-HMG-CoA reductase-1 included one hydrogen bond acceptor feature, three hydrophobic features, and fourteen exclusion volume spheres. To check the rationality of the HMG-CoA reductase pharmacophore model, the initial compound FBI was mapped with the best pharmacophore model. The mapping result was shown in Figure 3. The initial compound FBI was mapped well with all the features of the pharmacophore model of HMG-CoA reductase.

Three hydrogen bond acceptor features, one hydrogen bond donor feature, two hydrophobic features, and eighteen exclusion volume spheres composed the M-SQS-1. The initial compound D99 was mapped with the M-SQS-1. The methyl of the D99 was mapped with the hydrophobic feature. The hydroxyls in the aromatic ring were mapped with hydrogen accept features. The mapping result was shown in Figure 3. Three hydrophobic features, two hydrogen bond acceptor features, and one hydrogen bond donor feature formed the M-SREBP-2.

**Figure 4:**
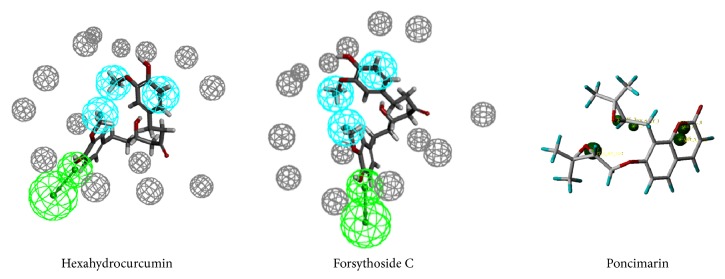
The mapping results of each TCM compound.

Hexahydrocurcumin was the potential compound with HMG-CoA reductase inhibition activity to build the component formula. Hexahydrocurcumin was mapped well with all the features of the HMG-CoA reductase pharmacophore model. The aromatic ring and the methyl in the aromatic ring were mapped with the hydrophobic features. The hydroxyl was mapped with the hydrogen bond acceptor feature. Forsythoside C was the potential inhibitor of the SQS to form the component formula. The mapping situation between the compound and the pharmacophore was shown in Figure 4. Forsythoside C has a satisfactory mapping situation with the SREBP-2 pharmacophore model.

### 3.3. Virtual Screening of the TCM Compounds

The best models were used to screen the TCMD. The numbers of compounds with potential activity of HMG-CoA reductase and drug-like properties were obtained was 2101. The number of SQS compounds was 1430 and 29 compounds of the SREBP-2 were screened. Then the compounds of HMG-CoA reductase and SQS would be further studied by molecular docking.

### 3.4. Molecular Docking

**Figure 5:**
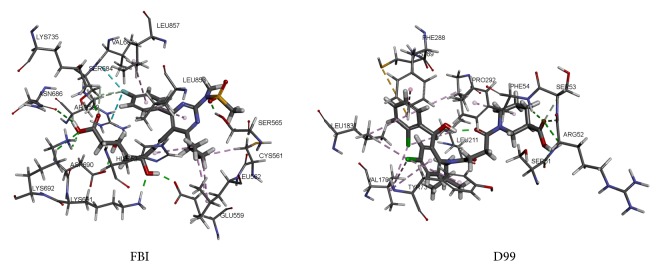
Interaction between the initial components and the HMG-CoA reductase and SQS.

The binding site of HMG-CoA reductase was defined with a radius of 8.54 Å, which was the same as the feature generation of pharmacophore. The CDOCKER method got a lower RMSD of 0.37 Å than 2.28 Å of the LibDock. Therefore, the CDOCKER method was chosen for the docking study of HMG-CoA reductase. The interaction of the initial compounds was shown in Figure 5. The initial compounds FBI generated the hydrophobic interactions with the residues CYS561, LEU562, HIS752, LEU853, and LEU857 and formed hydrogen bond interactions with the residues ARG590, ASP690, LYS691, LYS692, SER565, LYS735, ASN686, GLU559, and SER684. The docking of TCM compounds used the same parameter of initial compound.

The binding site of SQS was defined with a radius of 9.32 Å as the feature generation site of pharmacophore model. The RMSD of the CDOCKER was 0.77 Å, which was chosen for the docking study of SQS. The initial compound D99 generated hydrogen bond interactions in SER51, ARG52, SER53, and PHE54. Hydrophobic interactions existed in PHE54, TYR73, VAL179, LEU183, LEU211, PHE288, and PRO292 between D99 and protein. Besides, a Pi-Sulfur bond was generated in CYS289.

**Figure 6:**
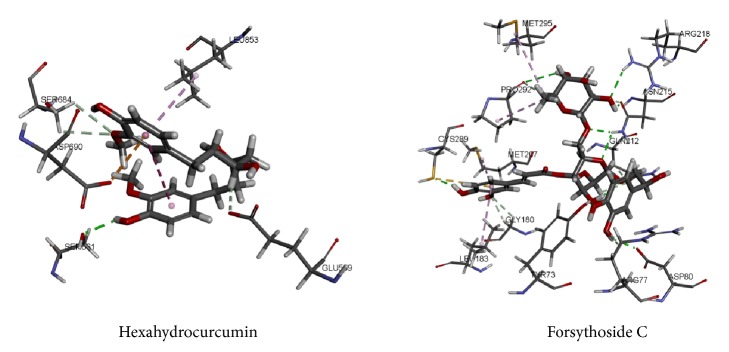
Interaction between the TCM components and the HMG-CoA reductase and SQS.

Hexahydrocurcumin formed hydrogen bond interactions with SER661, SER684, and GLU559 and formed hydrophobic interactions with LEU853, which has a similar binding mode to the initial compound. It had been reported that the fermented* Curcuma longa* has regulation of adipogenesis in high-fat diet-induced obese rats [45]. Besides, the extra of* Curcuma longa* lessens high-fat diet-induced inflammation in subcutaneous adipose tissue with the white pepper [46]. In the molecular docking, Forsythoside C had the hydrophobic bond interactions with PHE288, VAL179, LEU211, LEU183, PHE54, TYR73, and PRO292. LEU183 and PRO292 were also the key amino acids of the initial compound D99. Besides, Forsythoside C and D99 both formed the Pi-Sulfur bond in CYS289, which suggested that the Pi-Sulfur bond may be a necessary interaction between the compounds and SQS. Poncimarin from* Aurantii Fructus Immaturus *was chosen from the SREBP-2 result. The compound was mapping well with the pharmacophore.* Aurantii Fructus Immaturus *was a part of the formula with hypercholesterolemia treatment function [47]. The interaction of the TCM compounds was shown in Figure 6.

### 3.5. Biological Network Construction

**Figure 7:**
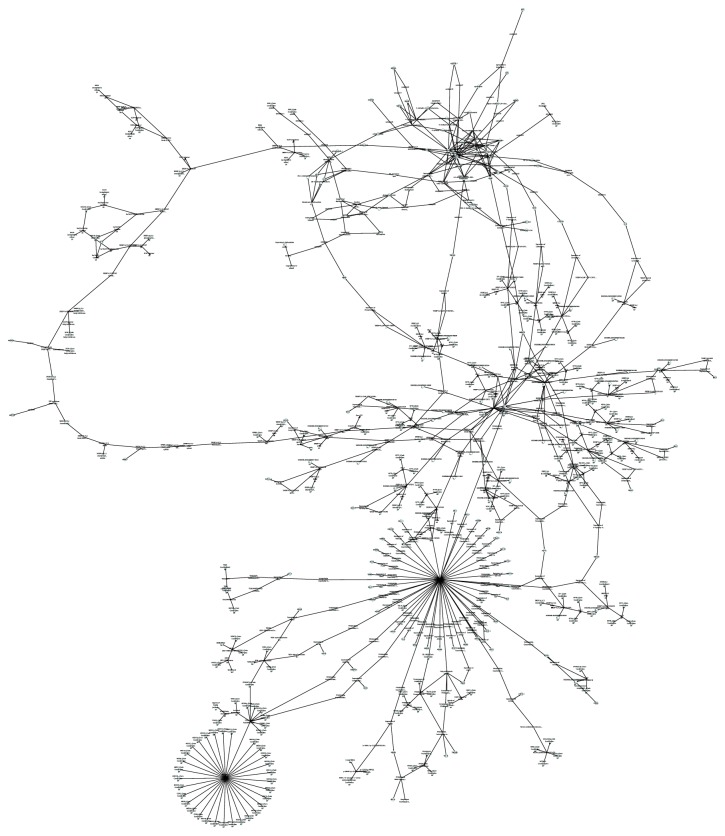
Metabolic network of the HMG-CoA reductase, SQS, and SREBP-2.

The metabolic network was constructed based on the pathways of HMG-CoA reductase, SQS, and SREBP-2 to elucidate the synergistic action mechanism of compounds. The network was shown in Figure 7. The parameters of the network were calculated to validate the stability and fault tolerance of the network and shown in Table 3.

The connectivity of the network was 100%, which proved the network with a fine connected condition. The network built in this research was in scale-less property because the value Y in the Barabási-Albert model was -1.557.

Through the analysis of parameter, stability and fault tolerance of network can be proven. Then the network was applied intuitively to show the interaction among the three targets and the mechanism of formula synergistic effect.

The network has shown a synergistic effect of the three targets. Cholesterol is generated by 25-step catalytic synthesis as end-product in the pathway. HMG-CoA reductase, an important rate-limiting enzyme in the synthesis process, plays an important role in catalysis to generate a mevalonic acid (MVA). MVA would generate farnesyl pyrophosphate (FPP) through several steps of catalytic synthesis reaction. FPP is the precursor of SQ. The generation of SQ takes place in the catalytic action of SQS. After several steps of enzymatic reaction, SQ eventually generates cholesterol. The inhibition of the enzyme activity can reduce the yield of catalysate. In the pathway, cholesterol levels would significantly reduce when the HMG-CoA reductase and SQS are both inhibited. While the genes expression of HMG-CoA reductase and SQS is regulated by SREBP-2, when SREBP-2 is restrained, HMG-CoA reductase and SQS transcription levels would decline. The reduction of HMG-CoA reductase and SQS transcription would reduce the enzymatic reaction rate. Combining the inhibition effect of three targets, the content of cholesterol would be reduced even more.

Besides, the network has shown that three targets would produce a better effect by affecting other important targets. Figure 7 had shown that the PPARs family was one of the important parts of the network. In this family, PPAR-alpha is one of the most important targets in the treatment of hypercholesterolemia. Its agonist, Fibrates, can reduce the levels of triglycerides by 30-50% [48]. They can also reduce LDL-C potentially. Besides, the levels of HDL-C increased by 5-15% depending on lipid phenotype and baseline concentration [49]. HDL is a significant protective factor in the plasma against coronary heart disease [50]. So the potential action of PPAR-alpha through the network may play an aided role in the overall effect of the component formula for a better effect.

### 3.6. The Design of TCM Component Formula

The information of the components was shown in Table 4.

The three compounds were chosen according to the criteria in Materials and Methods. The source plants of the compounds were commonly used plants in the TCM. The extra of* Curcuma longa* lessens high-fat diet-induced inflammation in subcutaneous adipose tissue with the white pepper [51].* Aurantii Fructus Immaturus* was a part of the formula with hypercholesterolemia treatment function [47].

**Figure 8:**
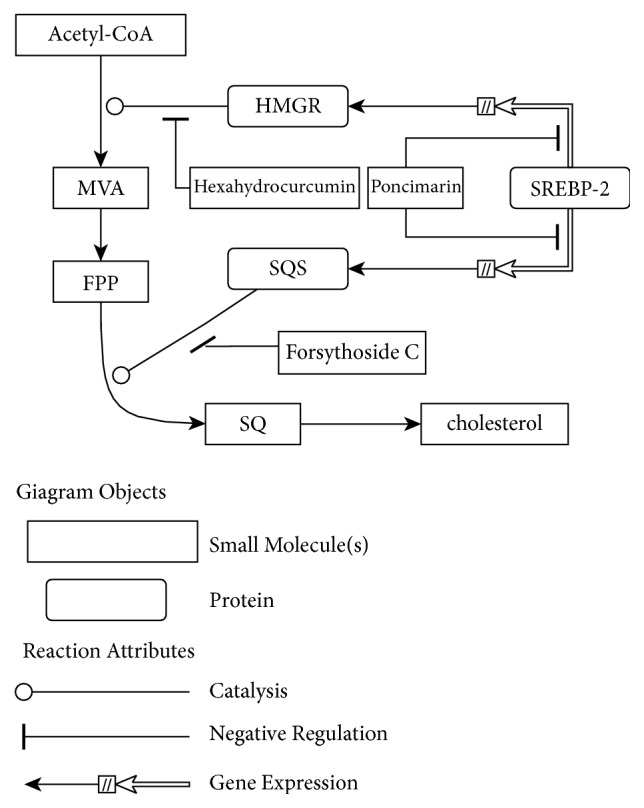
The synergistic effect of TCM component formula.

The combination of the three compounds had synergistic effect to decrease cholesterol levels, which was clearly illuminated by biological network. The mechanism action was separated from network model and shown in Figure 8. Hexahydrocurcumin and Forsythoside C reduce the cholesterol through inhibition of HMG-CoA reductase and SQS. Then poncimarin helps controlling the blood lipid by the PPARs family. Through synergistic effect of three compounds, the inhibition of this pathway can be enhanced by lowering enzyme activity and reducing the content of the enzyme so as to achieve the production of reinforcement in cholesterol synthesis inhibition.

### 3.7. The Activity of the TCM Compounds

**Figure 9:**
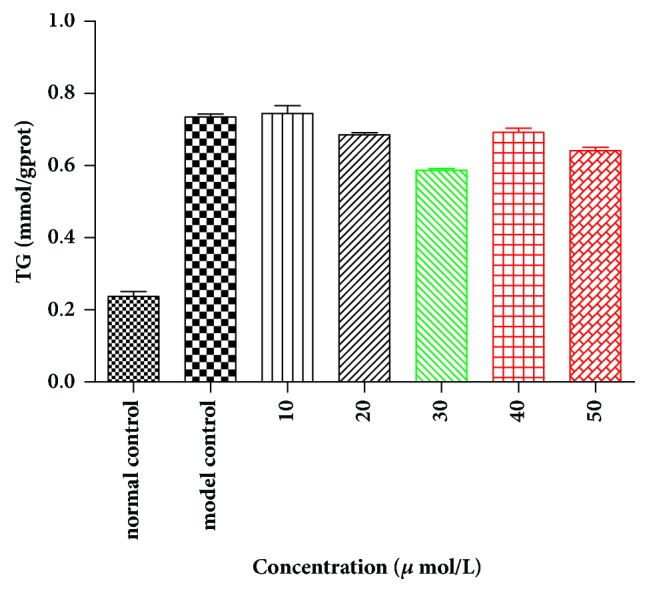
The TG-decrease effect of hexahydrocurcumin.

The test of lipid-lowering effect of hexahydrocurcumin (CAS number: 36062-05-2) with various doses was treated on cancer cell lines and HepG2 cell lines. Cell viability was examined by the MTT assay for 24 h. Then the total triglyceride (TG) was measured. The results indicated that the total triglyceride displayed a dose-dependent inhibition (10, 20, 30, 40, and 50 *μ*mol/L) with 20.11% decrease of TG level in 30 *μ*mol/L (Figure 9). The verification of the TCM compounds on targets level will be further studied.

## 4. Conclusion

This research built a TCM component formula formed by the TCM components acting on given targets chosen by virtual screen. A network was built for the interaction analysis. The component formula that contains poncimarin, hexahydrocurcumin, and Forsythoside C can get an enhanced lipid-lowering effect by the combined action of the targets. The component formula can enhance the inhibiting effect of the cholesterol synthesis.

This research hopes to provide a method to build a component formula based on components acting on the known targets. Through selection of targets, component formula can be designed. The components combine the advantages of the virtual screening and the TCM traditional action. Besides, other types of interaction can be shown in the network, which provided more design direction of component formula. Through the analysis of the component formula, the methods may provide a clue to the Traditional Chinese Medicine formula. The traditional formula may generate a complex interaction by the target which the component acted on.

## Figures and Tables

**Table 1:** The information of the structures of HMG-CoA reductase and SQS.

**PDB ID**	**Resolution (Å)**	**Ligand ID**	**I** **C**_50_ ** (nM)**	**Target**
3ASX	2.00	D99	20.0	SQS
1HWL	2.10	FBI	0.9	HMG-CoA reductase

**Table 2:** The validation results of each pharmacophore model.

**Name**	**Feature**	**Specificity**	**Energy**	**An** ^a^	**Dn** ^b^	**Ha** ^c^	**Ht** ^d^	***HRA*** ^e^	***IEI*** ^f^	***CAI*** ^g^
M-HMG-CoA reductase-1	AH_3_Ev_14_	—	—	92	375	52	72	0.56	2.94	1.66
M-HMG-CoA reductase-2	AH_3_Ev_14_	—	—	92	375	60	203	0.25	1.20	0.79
M-SQS-1	A_3_DH_2_Ev_18_	—	—	96	329	41	54	0.43	2.60	1.11
M-SQS-2	A_3_DH_2_Ev_18_	—	—	96	329	54	140	0.56	0.56	0.74
M-SREBP-1	A_2_DH_3_	3.7	6.26	20	60	20	40	100	2.00	2
M-SREBP-2	A_2_DH_3_	3.9	3.79	20	60	20	24	100	3.33	3.33
M-SREBP-3	A_2_DH_3_	4.6	5.17	20	60	20	22	100	3.64	3.64

^a^An: active compounds number. ^b^Dn: all compounds in test number. ^c^Ha: hit active compounds number. ^d^Ht: hit compounds number. ^e^*HRA*: the hit ratio of active compounds. ^f^*IEI*: identification of effective index. ^g^*CAI*: comprehensive appraisal index.

**Table 3:** Topological parameters of the network.

**Parameters**	**Network**	**Parameters**	**Network**
Nodes	705	Edges	659
Connected components	1	Network diameter	26
Network radius	15	Network centralization	0.065
Shortest paths	496320 (100%)	Characteristic path length	10.635
Average number of neighbors	2.357	Network heterogeneity	1.181

**Table 4:** Information of the potential active TCM components.

**Target**	**Name**	**Source Plant**
SREBP-2	Poncimarin	*Aurantii Fructus Immaturus* [44]
HMG-CoA reductase	Hexahydrocurcumin	*Zingiber officinale, Alpinia officinarum, Curcuma longa*
SQS	Forsythoside C	*Forsythia suspensa*

## References

[1] Goldstein J. L., Hazzard W. R., Schrott H. G., Bierman E. L., Motulsky A. G. (1973). Hyperlipidemia in coronary heart disease. I. Lipid levels in 500 survivors of myocardial infarction. *The Journal of Clinical Investigation*.

[2] Goldstein J. L., Schrott H. G., Hazzard W. R., Bierman E. L., Motulsky A. G. (1973). Hyperlipidemia in coronary heart disease. II. Genetic analysis of lipid levels in 176 families and delineation of a new inherited disorder, combined hyperlipidemia. *The Journal of Clinical Investigation*.

[3] Istvan E. S., Deisenhofer J. (2001). Structural mechanism for statin inhibition of HMG-CoA reductase. *Science*.

[4] Cannon C. P., Braunwald E., McCabe C. H. (2004). Intensive versus moderate lipid lowering with statins after acute coronary syndromes. *The New England Journal of Medicine*.

[5] Kureishi Y., Luo Z., Shiojima I. (2000). The HMG-CoA reductase inhibitor simvastatin activates the protein kinase Akt and promotes angiogenesis in normocholesterolemic animals. *Nature Medicine*.

[6] Endres M., Laufs U., Huang Z. (1998). Stroke protection by 3-hydroxy-3-methylglutaryl (HMG)-CoA reductase inhibitors mediated by endothelial nitric oxide synthase. *Proceedings of the National Acadamy of Sciences of the United States of America*.

[7] Pandit J., Danley D. E., Schulte G. K. (2000). Crystal structure of human squalene synthase. A key enzyme in cholesterol biosynthesis. *The Journal of Biological Chemistry*.

[8] Davidson M. H. (2007). Squalene synthase inhibition: A novel target for the management of dyslipidemia. *Current Atherosclerosis Reports*.

[9] Nishimoto T., Amano Y., Tozawa R. (2003). Lipid-lowering properties of TAK-475, a squalene synthase inhibitor, in vivo and in vitro. *British Journal of Pharmacology*.

[10] Hiyoshi H., Yanagimachi M., Ito M. (2000). Effect of ER-27856, a novel squalene synthase inhibitor, on plasma cholesterol in rhesus monkeys: Comparison with 3-hydroxy-3-methylglutaryl-CoA reductase inhibitors. *Journal of Lipid Research*.

[11] Flint O. P., Masters B. A., Gregg R. E., Durham S. K. (1997). Inhibition of cholesterol synthesis by squalene synthase inhibitors does not induce myotoxicity in vitro. *Toxicology and Applied Pharmacology*.

[12] Nishimoto T., Ishikawa E., Anayama H. (2007). Protective effects of a squalene synthase inhibitor, lapaquistat acetate (TAK-475), on statin-induced myotoxicity in guinea pigs. *Toxicology and Applied Pharmacology*.

[13] Burnett J. R. (2006). Drug evaluation: TAK-475 - An oral inhibitor of squalene synthase for hyperlipidemia. *Current Opinion in Infectious Diseases*.

[14] Horton J. D., Goldstein J. L., Brown M. S. (2002). SREBPs: activators of the complete program of cholesterol and fatty acid synthesis in the liver. *The Journal of Clinical Investigation*.

[15] Brown M. S., Goldstein J. L. (1997). The *SREBP* pathway: regulation of cholesterol metabolism by proteolysis of a membrane-bound transcription factor. *Cell*.

[16] Kong W., Wei J., Abidi P. (2004). Berberine is a novel cholesterol-lowering drug working through a unique mechanism distinct from statins. *Nature Medicine*.

[17] Grundy S. M., Vega G. L., Yuan Z., Battisti W. P., Brady W. E., Palmisano J. (2005). Effectiveness and tolerability of simvastatin plus fenofibrate for combined hyperlipidemia (the SAFARI trial). *American Journal of Cardiology*.

[18] Stein E. A., Davidson M. H., Dujovne C. A. (1996). Efficacy and tolerability of low-dose simvastatin and niacin, alone and in combination, in patients with combined hyperlipidemia: A prospective trial. *Journal of Cardiovascular Pharmacology and Therapeutics*.

[19] Verpoorte R., Crommelin D., Danhof M. (2009). Commentary: ‘A systems view on the future of medicine: inspiration from Chinese medicine?’. *Journal of Ethnopharmacology*.

[20] Liu X., Wang Q., Song G., Zhang G., Ye Z., Williamson E. M. (2014). The classification and application of toxic chinese materia medica. *Phytotherapy Research*.

[21] Klayman D. L. (1985). Qinghaosu (Artemisinin): An antimalarial drug from China. *Science*.

[22] Kołaczkowski M., Nowak M., Pawłowski M., Bojarski A. J. (2006). Receptor-based pharmacophores for serotonin 5-HT7R antagonists - Implications to selectivity. *Journal of Medicinal Chemistry*.

[23] Kim K.-H., Kim N. D., Seong B.-L. (2010). Pharmacophore-based virtual screening: A review of recent applications. *Expert Opinion on Drug Discovery*.

[24] Richmond N. J., Abrams C. A., Wolohan P. R. N., Abrahamian E., Willett P., Clark R. D. (2006). GALAHAD: 1. Pharmacophore identification by hypermolecular alignment of ligands in 3D. *Journal of Computer-Aided Molecular Design*.

[25] Chen C. Y.-C. (2009). Computational screening and design of traditional Chinese medicine (TCM) to block phosphodiesterase-5. *Journal of Molecular Graphics and Modelling*.

[26] Chen C.-Y., Chang Y.-H., Bau D.-T. (2009). Discovery of potent inhibitors for phosphodiesterase 5 by virtual screening and pharmacophore analysis. *Acta Pharmacologica Sinica*.

[27] Zhao J., Jiang P., Zhang W. (2009). Molecular networks for the study of TCM pharmacology. *Briefings in Bioinformatics*.

[28] Nakayama T., Suzuki S., Kudo H., Sassa S., Nomura M., Sakamoto S. (2007). Effects of three Chinese herbal medicines on plasma and liver lipids in mice fed a high-fat diet. *Journal of Ethnopharmacology*.

[29] Zhang G. B., Li Q. Y., Chen Q. L., Su S. B. (2013). Network pharmacology: a new approach for chinese herbal medicine research. *Evidence-Based Complementary and Alternative Medicine*.

[30] Barillari C., Marcou G., Rognan D. (2008). Hot-spots-guided receptor-based pharmacophores (HS-pharm): A knowledge-based approach to identify ligand-anchoring atoms in protein cavities and prioritize structure-based pharmacophores. *Journal of Chemical Information and Modeling*.

[31] Yang S.-Y. (2010). Pharmacophore modeling and applications in drug discovery: challenges and recent advances. *Drug Discovery Therapy*.

[32] Kim H. S., Ohno M., Xu B. (2003). 2-Substitution of adenine nucleotide analogues containing a bicyclo[3.1.0]hexane ring system locked in a northern conformation: enhanced potency as P2Y_1_ receptor antagonists. *Journal of Medicinal Chemistry*.

[33] Mustata G., Follis A. V., Hammoudeh D. I. (2009). Discovery of novel myc-max heterodimer disruptors with a three-dimensional pharmacophore model. *Journal of Medicinal Chemistry*.

[34] Guner O. F. (2000). *Pharmacophore Perception, Development, and Use in Drug Design*.

[35] Yang Z., Zhang Y., Wang X., Qiao Y. Pharmacophore model generation of P2Y 12 inhibitor.

[36] Zhou Z., Felts A. K., Friesner R. A., Levy R. M. (2007). Comparative performance of several flexible docking programs and scoring functions: Enrichment studies for a diverse set of pharmaceutically relevant targets. *Journal of Chemical Information and Modeling*.

[37] Bursulaya B. D., Totrov M., Abagyan R., Brooks C. L. (2003). Comparative study of several algorithms for flexible ligand docking. *Journal of Computer-Aided Molecular Design*.

[38] Croft D., O'Kelly G., Wu G. (2011). Reactome: a database of reactions, pathways and biological processes. *Nucleic Acids Research*.

[39] Shannon P., Markiel A., Ozier O. (2003). Cytoscape: a software Environment for integrated models of biomolecular interaction networks. *Genome Research*.

[40] Smoot M. E., Ono K., Ruscheinski J., Wang P. L., Ideker T. (2011). Cytoscape 2.8: new features for data integration and network visualization. *Bioinformatics*.

[41] Barabási A., Oltvai Z. N. (2004). Network biology: understanding the cell's functional organization. *Nature Reviews Genetics*.

[42] Caballero J. (2010). 3D-QSAR (CoMFA and CoMSIA) and pharmacophore (GALAHAD) studies on the differential inhibition of aldose reductase by flavonoid compounds. *Journal of Molecular Graphics and Modelling*.

[43] Wolber G., Seidel T., Bendix F., Langer T. (2008). Molecule-pharmacophore superpositioning and pattern matching in computational drug design. *Drug Discovery Therapy*.

[44] Guiotto A., Rodighiero P., Quintily U. (1975). Poncimarin, a new coumarin from Poncirus trifoliata L.. *Zeitschrift fur Naturforschung Section C Biosciences*.

[45] Kim J. H., Kim O.-K., Yoon H.-G. (2016). Anti-obesity effect of extract from fermented Curcuma longa L. through regulation of adipogenesis and lipolysis pathway in high-fat diet-induced obese rats. *Food and Nutrition Research*.

[46] Neyrinck A. M., Alligier M., Memvanga P. B. (2013). *Curcuma longa* extract associated with white pepper lessens high fat diet-induced inflammation in subcutaneous adipose tissue. *PLoS ONE*.

[47] Song X., Wang J., Wang P., Tian N., Yang M., Kong L. (2013). ^1^H NMR-based metabolomics approach to evaluate the effect of Xue-Fu-Zhu-Yu decoction on hyperlipidemia rats induced by high-fat diet. *Journal of Pharmaceutical and Biomedical Analysis*.

[48] Staels B., Dallongeville J., Auwerx J., Schoonjans K., Leitersdorf E., Fruchart J. (1998). Mechanism of action of fibrates on lipid and lipoprotein metabolism. *Circulation*.

[49] Freitas W. M., Quaglia L. A., Santos S. N. (2015). Low HDL cholesterol but not high LDL cholesterol is independently associated with subclinical coronary atherosclerosis in healthy octogenarians. *Aging Clinical and Experimental Research*.

[50] Gordon T., Castelli W. P., Hjortland M. C., Kannel W. B., Dawber T. R. (1977). High density lipoprotein as a protective factor against coronary heart disease. The Framingham study. *American Journal of Medicine*.

[51] Rana M., Reddy S. S., Maurya P. (2015). Turmerone enriched standardized Curcuma longa extract alleviates LPS induced inflammation and cytokine production by regulating TLR4-IRAK1-ROS-MAPK-NF*κ*B axis. *Journal of Functional Foods*.

